# Double puncta canaliculi may exhibit different clinical presentations

**DOI:** 10.5935/0004-2749.20200063

**Published:** 2020

**Authors:** Ipek Cigdem Ucar, Remzi Karadag

**Affiliations:** 1 Department of Ophthalmology, School of Medicine, Istanbul Medeniyet University, Istanbul, Turkey

**Keywords:** Eye abnormalities, Eye diseases, Eyelids/abnormalities, Lacrimal apparatus, Dacryocystorhinostomy, Anormalidades do olho, Doenças oculares, Pálpebras/anormalidades, Aparelho lacrimal, Dacriocistorinostomia

## Abstract

In this paper, we describe two adult patients who presented with double lacrimal puncta: one of them was asymptomatic and incidentally diagnosed, and the other complained of epiphora. In both patients, unilaterality, preference for the lower lid, and location medial to the normal punctum were common features of the accessory punctum. In the asymptomatic patient, irrigation revealed no obstruction in the punctum or the nasolacrimal drainage system. By contrast, the other patient’s nasolacrimal drainage system exhibited obstruction. Therefore, dacryocystorhinostomy surgery and silicone tube intubation were successfully performed. Double lacrimal puncta may be associated with epiphora or dry eye. These manifestations can easily be missed in a routine examination. This report was written to emphasize that unilateral epiphora of dry eye symptoms may be related to supernumerary punctum or canalicular systems and can easily be diagnosed with lid eversion.

## Abstract (pt)

Neste artigo, descrevemos dois pacientes adultos que apresentaram punção lacrimal dupla: um deles assintomático e diagnosticado incidentalmente, e o outro queixava-se de epífora. Nos dois pacientes, unilateralidade, preferência pela pálpebra inferior e posição medial pelo ponto normal foram características comuns do ponto acessório. No paciente assintomático, a irrigação não revelou obstrução no ponto ou no sistema de drenagem nasolacrimal. No entanto, o sistema de drenagem nasolacrimal do outro paciente exibiu obstrução. Portanto, a cirurgia de dacriocistorrinostomia e a intubação com tubo de silicone foram realizadas com sucesso. O ponto lacrimal duplo pode ser associado à epífora ou ao olho seco. Essas manifestações podem ser facilmente esquecidas em um exame de rotina. Queremos enfatizar que a epífora unilateral dos sintomas de olho seco pode estar relacionada ao sistema de ponto ou canalicular supranumerário e pode ser facilmente diagnosticada com eversão de pálpebra.

## INTRODUCTION

Supernumerary puncta, punctum duplication, and accessory punctum are all used to describe more than one lacrimal punctum, which is an infrequently observed congenital anomaly. Previous studies determined that the incidence of multiple puncta was 1/8001/60000^(1)^. An accessory punctum is usually located at the medial side of the lower punctum. Both puncta may have their own canaliculi, or one may be rudimentary. This condition is mostly asymptomatic but has also been reported to cause epiphora or dry eye^(2,3)^. In this report, we describe two patients who had different presenting symptoms of double lacrimal puncta.

## CASE REPORTS

### Case 1

**Figure 1:**
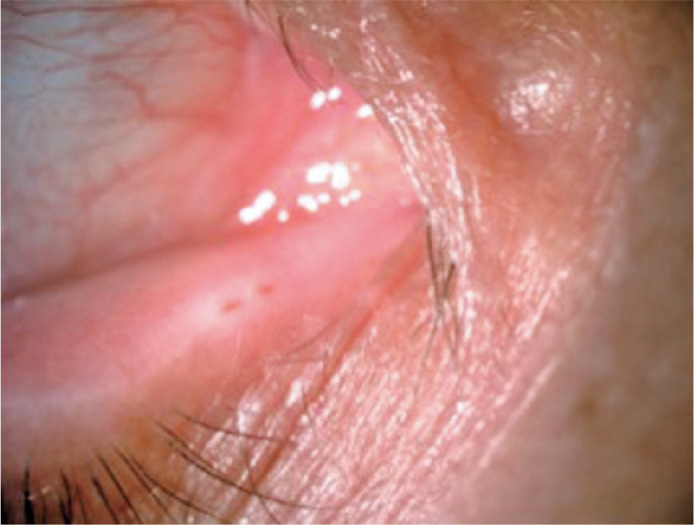
Case 1. Double lower eyelid puncta in the right eye.

A 61-year-old woman presented to our clinic for a routine ophthalmological examination. On slit-lamp examination, double puncta were found on the right lower lid: one was in a normal position, and the other was located 1 mm medial to the first punctum. Both puncta had a normal appearance (Figure 1), the size of the lateral punctum was 1 mm, and the size of the medial punctum was 0.8 mm. Irrigation with fluid showed that both puncta separately communicated with the lacrimal sac through separate canaliculi. Irrigation of both lower puncta and the upper punctum revealed no obstruction in the drainage system. The upper punctum of the right eye and both upper and lower puncta and the drainage system of the left eye were normal.

### Case 2

**Figure 2:**
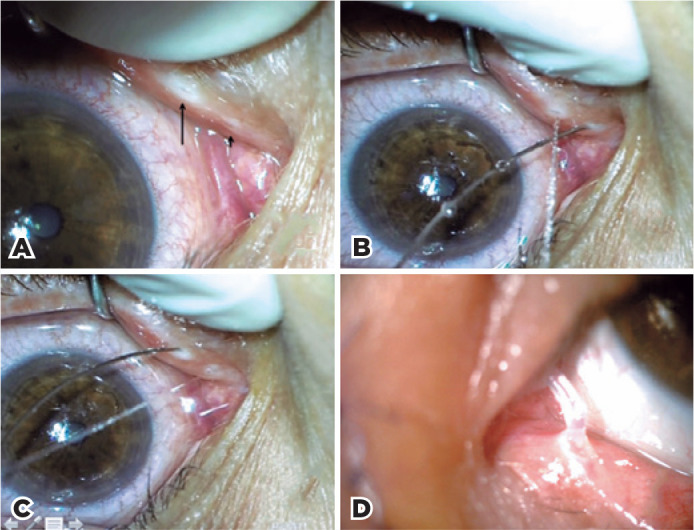
Case 2. A) Double lower eyelid puncta in the left eye. B, C) The fluid was injected through one punctum and ejected through the other punctum. D) Dacryocystorhinostomy surgery and silicone tube intubation were successfully performed (image collected at 1 week postoperatively).

A 43-year-old woman presented with a 10-year history of tearing in her left eye. Examination revealed two puncta on the left lower lid: one had a normal appearance and was located in a normal position, and the other had a rudimentary appearance and was situated 2 mm medial to the normal punctum with a slit configuration (Figure 2A). The size of the normal punctum was 1.1 mm, whereas the size of the accessory punctum was 0.4 mm. Irrigation of the normal punctum revealed that the flow of fluid was ejected through the accessory punctum, and irrigation of the accessory punctum revealed that the flow of fluid was ejected through the normal punctum (Figures 2B-2C). Furthermore, injection of fluid into the upper punctum of the left eye revealed that there was an obstruction in the left nasolacrimal drainage system. The upper punctum of the left eye and both upper and lower puncta and the drainage system of the right eye were normal. Dacryocystorhinostomy surgery and silicone tube intubation were successfully performed on the left eye (Figure 2D), and the patient did not exhibit epiphora during 1 month of follow-up.

Neither patient had a history of trauma or previous surgery. Written informed consent was obtained from both patients for publication of their clinical information.

## DISCUSSION

The nasolacrimal drainage system originates from surface ectoderm. Incomplete separation of the core from the surface epithelium and abnormal budding of the epithelial cord are the presumed causes of anomalies in this system^(4)^. Previous studies reported that the characteristic features of accessory puncta were unilaterality, preference for the lower lid, and location medial to the normal punctum^(5-7)^. Our cases are similar to previous case series with respect to these features. The effects of an additional lower punctum and canaliculus on lacrimal drainage are not well known. In many publications, such manifestations have been described as incidental examination findings in asymptomatic individuals^(2,5-7)^. However, Satchi et al. presented the largest series of patients with supernumerary puncta, consisting of 23 patients^(2)^. In their study, the presence of double puncta was an incidental examination finding in only five patients, whereas 18 patients complained of epiphora on the same side as the double puncta.

Epiphora (“dry eye”) has been previously documented in patients with accessory puncta^(1-3)^. The occurrence of epiphora in childhood may be related to congenital anomalies of the nasolacrimal system^(2,8)^. In elderly patients, epiphora may be associated with a dysfunction of the lacrimal drainage system, defined as functional epiphora, or acquired nasolacrimal duct obstruction^(2,9)^. In the patient in case 2, the underlying reason for epiphora was complete nasolacrimal duct obstruction; thus, the patient underwent dacryocystorhinostomy surgery.

In the study by Satchi et al., 5 of 18 patients with epiphora had complete nasolacrimal duct obstruction, and the remaining 13 patients had either partial nasolacrimal duct obstruction or functional epiphora. In another study that included 12 adult patients with double puncta, Bacskulin found that all 12 complained of epiphora, although they had a patent lacrimal system^(9)^.

Bair et al. reported that faster tear drainage was the causal mechanism of dry eye^(3)^. The presence of an accessory punctum and canaliculus does not always result in increased drainage. Kakizaki et al. suggested a two-compartment model for the lacrimal canalicular drainage system^(10)^, which explains why some patients with supernumerary puncta exhibit tearing and other patients are asymptomatic. The association of the accessory canaliculus with its origin and the Horner’s muscle (the lacrimal component of the orbicularis oculi) may determine the direction of tear flow within the accessory canaliculus and the effect on canalicular function and related symptoms^(10)^.

Here, the patient in case 1 was asymptomatic, and no intervention was performed. The patient was simply informed of her condition. The patient in case 2 underwent dacryocystorhinostomy surgery due to epiphora and complete nasolacrimal duct obstruction. In the study by Satchi et al., of nine adults who underwent dacryocystorhinostomy surgery, five had complete nasolacrimal duct obstruction, two had partial nasolacrimal duct obstruction, and two had freely patent lacrimal systems^(2)^. Although dacryocystorhinostomy is performed because of nasolacrimal duct obstruction, it can also be performed because of dysfunctional drainage. Successful results have been obtained in patients who exhibit this type of dysfunction^(2)^. It should be considered that, in patients who exhibit epiphora in adulthood, dysfunction of the lacrimal drainage system and acquired nasolacrimal duct obstruction may contribute to the onset of epiphora.

In conclusion, a healthy, working lacrimal drainage system has an important effect on ocular surface lubrication. Supernumerary puncta and canaliculi can easily be missed during the course of a routine examination. Patients who present with unilateral epiphora or dry eye symptoms, such as punctal apposition and abnormalities, should be evaluated using lid eversion during slit-lamp examination.
